# 30-Year Trends in Stroke Rates and Outcome in Auckland, New Zealand (1981-2012): A Multi-Ethnic Population-Based Series of Studies

**DOI:** 10.1371/journal.pone.0134609

**Published:** 2015-08-20

**Authors:** Valery L. Feigin, Rita V. Krishnamurthi, Suzanne Barker-Collo, Kathryn M. McPherson, P. Alan Barber, Varsha Parag, Bruce Arroll, Derrick A. Bennett, Martin Tobias, Amy Jones, Emma Witt, Paul Brown, Max Abbott, Rohit Bhattacharjee, Elaine Rush, Flora Minsun Suh, Alice Theadom, Yogini Rathnasabapathy, Braden Te Ao, Priya G. Parmar, Craig Anderson, Ruth Bonita, ARCOS IV Group

**Affiliations:** 1 Auckland University of Technology, Auckland, New Zealand; 2 The University of Auckland, Auckland, New Zealand; 3 Health Research Council of New Zealand, Auckland, New Zealand; 4 Nuffield Department of Population Health, University of Oxford, Oxford, United Kingdom; 5 Public Health Intelligence, Ministry of Health, Wellington, New Zealand; 6 University of California Merced, Merced, California, United States of America; 7 University of Queensland, Brisbane, Australia; 8 Waitemata District Health Board, North Shore Hospital, Auckland, New Zealand; 9 The University of Sydney, Camperdown, NSW, Australia; Oxford University, UNITED KINGDOM

## Abstract

**Background:**

Insufficient data exist on population-based trends in morbidity and mortality to determine the success of prevention strategies and improvements in health care delivery in stroke. The aim of this study was to determine trends in incidence and outcome (1-year mortality, 28-day case-fatality) in relation to management and risk factors for stroke in the multi-ethnic population of Auckland, New Zealand (NZ) over 30-years.

**Methods:**

Four stroke incidence population-based register studies were undertaken in adult residents (aged ≥15 years) of Auckland NZ in 1981–1982, 1991–1992, 2002–2003 and 2011–2012. All used standard World Health Organization (WHO) diagnostic criteria and multiple overlapping sources of case-ascertainment for hospitalised and non-hospitalised, fatal and non-fatal, new stroke events. Ethnicity was consistently self-identified into four major groups. Crude and age-adjusted (WHO world population standard) annual incidence and mortality with corresponding 95% confidence intervals (CI) were calculated per 100,000 people, assuming a Poisson distribution.

**Results:**

5400 new stroke patients were registered in four 12 month recruitment phases over the 30-year study period; 79% were NZ/European, 6% Māori, 8% Pacific people, and 7% were of Asian or other origin. Overall stroke incidence and 1-year mortality decreased by 23% (95% CI 5%-31%) and 62% (95% CI 36%-86%), respectively, from 1981 to 2012. Whilst stroke incidence and mortality declined across all groups in NZ from 1991, Māori and Pacific groups had the slowest rate of decline and continue to experience stroke at a significantly younger age (mean ages 60 and 62 years, respectively) compared with NZ/Europeans (mean age 75 years). There was also a decline in 28-day stroke case fatality (overall by 14%, 95% CI 11%-17%) across all ethnic groups from 1981 to 2012. However, there were significant increases in the frequencies of pre-morbid hypertension, myocardial infarction, and diabetes mellitus, but a reduction in frequency of current smoking among stroke patients.

**Conclusions:**

In this unique temporal series of studies spanning 30 years, stroke incidence, early case-fatality and 1-year mortality have declined, but ethnic disparities in risk and outcome for stroke persisted suggesting that primary stroke prevention remains crucial to reducing the burden of this disease.

## Introduction

The burden of stroke is large and increasing worldwide, with notable ethnic/racial disparities.[1–7] Effective primary stroke prevention strategies are therefore critical in ageing populations[1,8] and where there is increasing numbers of people surviving with stroke-related disability and ongoing risk.[9] However, because of the considerable challenges to determining temporal trends in incidence and outcome of stroke, there is limited information on the impact of declines (or increases) in rates and case fatality in whole populations. This leaves uncertainties regarding the impact of public health policies and improvements in health service delivery for this important disease.[10–13]

As a large proportion of the burden of stroke is borne outside the hospital sector, it is crucial that incident cases are ascertained and studied in a population-wide context.[10,11,13] Over the last two decades new primary and secondary preventative strategies have been implemented but the effect of the strategies on stroke burden have not been reliably assessed.[14] Accurate population-based data on population trends in stroke incidence and risk factors reflect the success or otherwise of prevention strategies, while case-fatality and mortality trends reflect management and natural history of stroke. All these indices are crucial for evidence-based planning and resource allocation for stroke services and preventive strategies. Yet population-based stroke incidence studies are complex,[10,13] and are limited in number when compared to those using hospital-based registers, mortality data, or studies limited to certain age groups. To address this gap, we used population-based data from the four Auckland Regional Community Stroke (ARCOS I—IV) studies (1981–1982, 1991–1992, 2002–2003 and 2011–2012) to determine 30-year trends in stroke incidence, case-fatality and mortality in the four major ethnic groups (NZ European, Asian, Māori and Pacific Islanders) in Auckland, New Zealand (NZ).

## Materials and Methods

### Study populations, case ascertainment and diagnostic criteria

The study population and methods of case ascertainment in the four ARCOS studies are described in detail elsewhere.[12,15–18] In brief, these studies utilised harmonised population-based registers of all new cases of stroke in the greater Auckland region over consistent 12 month calendar periods (total resident population aged ≥15 grew from 596,580 in 1981–1982 to 1,119,192 in 2011–2012). Multiple overlapping methods of case ascertainment, and standard WHO clinical diagnostic criteria for stroke were utilised.[19] For the 1991–1992 ARCOS study, all hospital-managed stroke events in the region, and a cluster survey of 25% of all primary care general practitioners (GPs) records, were used to estimate the total number of non-hospitalised, non-fatal stroke events.[12] For the 1981–1982 ARCOS study, a cluster sample of 50% of all registered GPs was used to identify a representative sample of stroke events in the study population. All deceased cases were identified through hospital admissions and discharge reports, systematic searches of post-mortem reports and death certificates in national registries. Data for fatal non-hospitalised SAH cases were collected from medical records only. In the two most recent studies (2002–2003 and 2011–2012)[12,15] no cluster sampling was used and instead the whole study population was monitored for new stroke events. To ensure a complete prospective case-ascertainment in 2002–2003 and 2011–2012 studies we undertook daily searches of hospital presentation data, where a diagnosis suggesting stroke or transient ischaemic attack (TIA) was recorded for all public hospitals and emergency departments, CT/MRI records and hospital discharge registers; weekly checks of all private hospitals, rest homes, and community health services (general practices, hospital outpatient clinics and rehabilitation centres); quarterly checks of coroner/autopsy records, death certificates (from Registrar of Births, Deaths and Marriages) to identify people who had died with any mention of stroke, and NZ Health Information Service data of all fatal and non-fatal stroke/TIA cases in the study population. In 2002–2003 and 2011–2012 studies strokes were subdivided into pathological types (ischaemic stroke [IS], primary intracerebral haemorrhage [PICH], subarachnoid haemorrhage [SAH]) according to neuroimaging (CT, MRI, or necropsy) findings. SAH was defined as “an abrupt onset of a severe headache and/or impaired consciousness or focal neurological signs associated with at least one of the following findings: uniform blood staining of the cerebrospinal fluid; CT evidence of blood in the subarachnoid space; cerebral angiographic identification of an aneurysm or arteriovenous malformation, or identification of SAH at surgery or at autopsy. This definition excludes PICH with extension into the subarachnoid space and subarachnoid bleeding due to trauma, neoplasms, or infections”.[20] All new stroke cases, including suspected strokes, were ascertained by study researchers across all four studies. Consistent diagnostic criteria were used across all studies to allow valid comparisons. Cases without imaging or pathological necropsy confirmation of stroke type were classified as stroke of undetermined type (undetermined). Cardiovascular risk factors and medication use were ascertained via medical records across all studies. All study participants were followed up for 1 year for fatal/non-fatal outcomes. Ethnicity was identified by self-report across all four studies. Regional Ethics Committees approved all four studies.

### Statistical analyses

For the 1981–1982 and 2002–2003 ARCOS studies participants selected one self-identified ethnicity to be used for the analyses. For the 1991–1992 and 2011–2012 ARCOS studies, multiple self-identified ethnicities were recorded and ethnicity was prioritised corresponding to the allocation process and using NZ census definitions for Māori, Pacific, Asian/other (“other” included ethnic groups such as other European, Middle Eastern, Latin American and African ethnicities) and NZ Europeans as per the NZ Census.[21] When multiple ethnicities were self-identified, ethnicity was prioritized as a single ethnic group for the purposes of analysis in the following order: Māori, Pacific, Asian/other, NZ European. For example if a person self-identified as NZ European and Māori, they were classified as Māori, and if they self-identified as Pacific and Chinese they were classified as Pacific. Ethnicity information was not available for 2 of the ARCOS 2011–2012 study participants and 46 of the ARCOS 2002–2003 study participants, who were excluded from the ethnicity analyses. Age, sex and ethnic structure of the corresponding Auckland census data were used as the denominator in calculating incidence.

Crude annual age-, sex-, and ethnic-specific stroke incidence (first-ever-in-a-lifetime events) and attack rates (all events, including recurrent strokes), 28-day case fatality (proportion [%] of people with stroke who died within 28 days of stroke onset among the total number of people with incident stroke) and mortality rates (number of people with incident stroke who died in the study population over one-year follow-up period [nominator] divided by the study population at risk [denominator]), with 95% confidence intervals (CI) per 100,000 people were calculated assuming a Poisson distribution. Sampling procedure differences between ARCOS 1981–1982 and 1991–1992 studies were taken into account when computing CI and standard error (SE) of all estimated rates, as described elsewhere.[12] Standardised rates were calculated using the direct method and age-standardised to WHO ‘World’ standard population.[22] Rate ratios (RR) were calculated to evaluate differences in age-adjusted rates between the 1981–1982 and 2011–2012 study periods, and the Wald statistic test of heterogeneity across age groups were performed.[12] For categorical variables, the Cochrane-Armitage test was used to assess whether there was evidence of a statistically significant trend. For continuous variables, analysis of variance or the Kruskal-Wallis test was used.

Completeness of case ascertainment based on the sources of notification was determined using capture-recapture techniques.[23] This involved conducting log-linear modelling assuming a Poisson distribution (unadjusted for sample procedures),[24] and used the four main sources of notification: hospital, general practitioner, death certificate and other sources. The final model with the least deviance, (deviance is an indicator of ‘goodness of fit’ and a lower deviance suggests a better fitting model), for all studies included the main effects of the four sources and the 3-way interaction between hospital, general practitioner and death certificate. A Bonferroni-corrected threshold of p = 0.00013 (based on 380 tests conducted 0.05/380) was used to indicate statistical significance after adjusting for multiple testing.

To compare our findings with similar population-based studies carried out in other countries we searched Medline with the terms”stroke”, “epidemiology”, “incidence”, “ethnic/racial”, “trend(s)”, and “population or community based” for population-based studies of stroke carried out between 1980 and 2014 and published in English. Only population-based studies from high-income countries that reported age-specific raw numbers of first-ever-in-a-lifetime strokes (numerator and populations at risk (denominator) sufficient to calculate incidence rates age-standardised to WHO ‘World’ standard population[22] were included in the analysis. We also compared trends in stroke incidence and mortality rates from our series of Auckland Regional Community Stroke (ARCOS) studies (1981–1982, 1991–1992, 2002–2003, 2011–2012) with those found in the Global Burden of Disease (GBD) 2010 study.[1,25]

## Results and Discussion

### Results

There were 5400 new stroke patients (47% men) registered across the four studies (Tables 1 and 2). Over the 30-year study period, the proportion of patients presenting with recurrent strokes decreased by 2.95% (95% CI 0.60%-5.83%) from 24.3% to 21.4%. The mean age of individuals with stroke increased (on average by 3 years) across ethnic groups, except for Asian/other ethnic group where age decreased by 4.6 years (95% CI 3.8–14.1). However, the 15-year gap between in the mean age at stroke onset in Māori and Pacific (youngest age group) compared with the mean age of stroke in NZ European (oldest age group) persisted over the 30 years. Although NZ European patients constituted the largest proportion of strokes, their proportional frequency reduced by 23% (95% CI 21.0%-26.0%) over the same period, while the proportional frequency of stroke patients from all other ethnic groups increased, with the largest increase being 8-fold for the Asian/other ethnic groups (Table 1).

**Table 1:** Baseline characteristics and 28-day outcome of stroke events in each ARCOS register.

	1981–1982	1991–1992	2002–2003	2011–2012	*P* for trend^§§^
	n (%)	n (%)	n (%)	n (%)	
**Demographics**					
Male	662 (48.7)	817 (46.4)	892 (46.0)	1012 (48.3)	0.977
Age, mean (±SD), years***					
NZ/European	72.2 (12.8)	73.5 (12.1)	75.6 (12.5)	75.3 (13.4)	<0.0001^§^
Māori	56.7 (14.2)	55.0 (16.1)	60.7 (14.3)	59.6 (15.5)	0.04
Pacific	55.8 (9.0)	59.7 (15.0)	64.5 (13.6)	61.6 (14.9)	0.002
Asian/other	72.1 (12.8)	65.6 (13.2)	65.9 (13.9)	67.5 (13.3)	0.199
Overall	71.2 (13.3)	71.6 (13.5)	73.0 (13.8)	71.6 (14.9)	0.001
Ethnicity					
NZ/European	1248 (91.8)	1532 (87.0)	1431 (75.6)	1434 (68.5)	<0.0001** ^§^
Māori	60 (4.4)	82 (4.7)	102 (5.4)	138 (6.6)	
Pacific	32 (2.4)	111 (6.3)	197 (10.4)	270 (12.9)	
Asian/other	20 (1.5)	36 (2.0)	162 (8.6)	252 (12.0)	
**Source of notification**					<0.0001** ^§^
Hospital	1082 (80.0)	1092 (62.0)	1361 (70.2)	1733 (82.7)	
General practitioner	30 (2.2)	368 (20.9)	117 (6.0)	2 (0.1)	
Death certificate	104 (7.7)	161 (9.1)	95 (4.9)	15 (0.7)	
Other sources	136 (10.1)	140 (8.0)	365 (18.8)	346 (16.5)	
**Premorbid risk factors (from medical notes)**					
Current smoking					
NZ/European	330 (26.7)	330 (21.7)	162 (12.6)	178 (12.8)	<0.0001^§^
Māori	32 (53.3)	41 (50.6)	35 (38.9)	55 (40.4)	0.159
Pacific	12 (37.5)	31 (28.7)	23 (13.1)	65 (24.4)	0.001
Asian/other	0	9 (25.0)	15 (10.3)	23 (9.3)	0.013
Overall	374 (27.7)	411 (23.5)	241 (14.0)	322 (15.8)	<0.0001^§^
High blood pressure					
NZ/European	632 (51.1)	802 (52.7)	783 (57.7)	947 (66.0)	<0.0001^§^
Māori	38 (63.3)	41 (52.6)	63 (62.4)	85 (61.6)	0.690
Pacific	22 (68.8)	49 (45.0)	124 (65.6)	178 (65.9)	0.022
Asian/other	8 (40.0)	18 (50.0)	88 (58.7)	184 (73.0)	<0.0001^§^
Overall	700 (51.5)	910 (52.1)	1079 (59.0)	1394 (66.5)	<0.0001^§^
Myocardial infarction					
NZ/European	146 (11.8)	273 (17.9)	190 (13.5)	401 (28.0)	<0.0001^§^
Māori	8 (13.8)	9 (11.4)	12 (11.9)	25 (18.1)	0.266
Pacific	2 (6.7)	3 (2.8)	15 (7.9)	34 (12.6)	0.004
Asian/other	0	3 (8.3)	17 (11.0)	47 (18.7)	0.003
Overall	156 (11.5)	288 (16.5)	240 (12.7)	507 (24.2)	<0.0001^§^
Previous stroke					
NZ/European	314 (25.3)	404 (26.4)	361 (25.5)	308 (21.5)	0.015
Māori	14 (23.3)	21 (25.6)	12 (11.9)	21 (15.2)	0.041
Pacific	2 (6.3)	25 (22.5)	54 (27.8)	62 (23.2)	0.234
Asian/other	0	6 (16.7)	36 (23.1)	56 (22.3)	0.067
Overall	330 (24.3)	456 (25.9)	477 (25.1)	448 (21.4)	0.016
Diabetes mellitus					
NZ/European	98 (7.9)	193 (12.6)	179 (12.7)	236 (16.5)	<0.0001^§^
Māori	20 (33.3)	19 (24.4)	35 (34.7)	41 (29.7)	0.975
Pacific	12 (46.2)	16 (15.0)	69 (36.1)	117 (43.3)	0.0003
Asian/other	4 (20.0)	8 (22.2)	40 (26.1)	77 (30.6)	0.131
Overall	134 (10.0)	236 (13.6)	329 (17.4)	471 (22.5)	<0.0001^§^
Atrial fibrillation					
NZ/European	NA	NA	328 (23.7)	460 (32.1)	<0.0001^§^
Māori	NA	NA	29 (28.7)	42 (30.4)	0.774
Pacific	NA	NA	41 (21.5)	62 (23.0)	0.704
Asian/other	NA	NA	18 (11.9)	47 (18.7)	0.075
Overall	NA	NA	416 (22.0)	611 (29.2)	<0.0001^§^
**Premorbid medication**					
Blood pressure lowering meds					
NZ/European	428 (35.2)	539 (35.2)	712 (50.8)	910 (63.5)	<0.0001^§^
Māori	26 (43.3)	21 (25.6)	49 (48.5)	79 (57.2)	0.001
Pacific	16 (50.0)	30 (27.0)	94 (49.2)	168 (62.2)	<0.0001^§^
Asian/other	6 (30.0)	14 (38.9)	73 (47.1)	163 (64.7)	<0.0001^§^
Overall	476 (35.0)	604 (34.3)	942 (49.0)	1321 (63.0)	<0.0001^§^
Antiplatelet agents					
NZ/European	314 (27.4)	358 (23.4)	665 (48.2)	707 (49.3)	<0.0001^§^
Māori	12 (21.4)	7 (8.5)	29 (29.6)	60 (43.5)	<0.0001^§^
Pacific	8 (28.6)	14 (12.6)	63 (33.5)	115 (42.6)	<0.0001^§^
Asian/other	4 (25.0)	8 (22.2)	56 (36.6)	117 (46.4)	0.001
Overall	338 (24.8)	387 (22.0)	832 (42.9)	999 (47.7)	<0.0001^§^
Anticoagulants					
NZ/European	NA	33 (2.2)	137 (9.9)	117 (8.2)	<0.0001^§^
Māori	NA	10 (12.2)	12 (12.2)	7 (5.1)	0.052
Pacific	NA	2 (1.8)	27 (14.1)	24 (8.9)	0.172
Asian/other	NA	0	8 (5.2)	14 (5.6)	0.269
Overall	NA	45 (2.6)	185 (9.9)	162 (7.7)	<0.0001^§^
Lipid lowering drugs					
NZ/European	NA	NA	213 (15.6)	567 (39.5)	<0.0001^§^
Māori	NA	NA	13 (13.4)	58 (42.0)	<0.0001^§^
Pacific	NA	NA	19 (10.4)	117 (43.3)	<0.0001^§^
Asian/other	NA	NA	27 (18.0)	116 (46.0)	<0.0001^§^
Overall	NA	NA	272 (14.4)	858 (40.9)	<0.0001^§^
**Management**					
Admission to hospital within 28 days of stroke onset					
NZ/European	768 (61.5)	1088 (71.0)	1283 (89.7)	1291 (90.0)	<0.0001^§^
Māori	46 (76.7)	73 (89.0)	99 (97.1)	124 (89.9)	0.021
Pacific	22 (68.8)	87 (78.4)	188 (95.4)	259 (95.9)	<0.0001^§^
Asian/other	14 (70.0)	28 (77.8)	157 (96.9)	230 (91.3)	0.010
Overall	850 (62.5)	1276 (72.5)	1727 (91.3)	1904 (90.9)	<0.0001^§^
Admission to acute stroke unit					
NZ/European	NA	NA	140 (9.8)	709 (50.8)	<0.0001^§^
Māori	NA	NA	32 (31.4)	61 (46.2)	0.021
Pacific	NA	NA	39 (19.8)	140 (54.1)	<0.0001^§^
Asian/other	NA	NA	24 (14.8)	131 (54.1)	<0.0001^§^
Overall	NA	NA	238 (12.3)	1041 (51.3)	<0.0001^§^
Neuroimaging, CT/MRI					
NZ/European	134 (18.9)	429 (38.9)	1236 (86.4)	1389 (97.1)	<0.0001^§^
Māori	18 (50.0)	50 (67.6)	99 (97.1)	134 (97.8)	<0.0001^§^
Pacific	6 (42.9)	50 (57.5)	180 (91.4)	261 (97.4)	<0.0001^§^
Asian/other	4 (40.0)	12 (42.9)	153 (95.0)	244 (96.8)	<0.0001^±^
Overall	162 (11.9)	541 (41.9)	1694 (87.6)	2030 (97.2)	<0.0001^§^
**28-day case-fatality**					
NZ/European	408 (32.7)	362 (23.6)	304 (21.2)	279 (19.5)	<0.0001^§^
Māori	18 (30.0)	20 (24.4)	24 (23.5)	23 (16.7)	0.033
Pacific	14 (43.8)	32 (28.8)	39 (19.8)	43 (15.9)	<0.0001^§^
Asian/other	10 (50.0)	7 (19.4)	24 (14.8)	47 (18.7)	0.056
Overall	450 (33.1)	421 (23.9)	407 (20.7)	393 (18.8)	<0.0001^§^
**Pathological type of stroke**					
Ischaemic stroke	NA	NA	1380 (71.2)	1694 (80.8)	<0.0001^§^
Primary intracerebral haemorrhage	NA	NA	236 (12.2)	275 (13.1)	0.368
Subarachnoid haemorrhage	90 (6.6)	76 (4.3)	96 (5.0)	87 (4.2)	0.008
Undetermined	NA	NA	226 (11.7)	40 (1.9)	<0.0001^§^
**Time from stroke onset to study assessment (days), median (interquartile range)** *					
NZ/European	19 (6–38)	17 (7–64)	27 (5–129)	6 (3–27)	<0.0001^§^
Māori	31 (5–42)	18 (6–77)	25 (4–112)	7 (3–91)	0.097
Pacific	21 (9–58)	13 (6–57)	15 (4–132)	5 (2–27)	<0.0001^§^
Asian/other	29 (7–44)	12 (5–67)	18 (5–115)	6 (3–28)	<0.0001^§^
Overall	20 (6–38)	16 (6–64)	27 (5–139)	6 (3–29)	<0.0001^§^
**Capture-recapture, missing** ****	131/677 (19.3)	185/1449 (12.8)	144/1938 (7.4)	30/2096 (1.4)	<0.0001^§^

^§§^ *P* value calculated using Cochrane-Armitage trend test;

*P value calculated using Kruskal-Wallis test;

**P value calculated using Chi-squared test,

***P value calculated using analysis of variance test,

**** Using log-linear model containing main effects and interaction for hospital x general practitioner x death certificate. Information about medication was collected in a month prior to stroke.

^§^ *P*-values that remained significant after applying Bonferroni-correction to adjust for multiple testing for 380 tests (19 variables [age, source of notification, high blood pressure, myocardial infarction, previous stroke, diabetes mellitus, atrial fibrillation, current smoking, blood pressure lowering medication, antiplatelet agents, anticoagulants, lipid lowering drugs, admission to hospital within 28 days of stroke onset, admission to acute stroke unit, neuroimaging/CT/MRI, 28-day case fatality, pathological type of stroke, time from stroke onset, capture-recapture) by 5 ethnic grouping (New Zealand European, Maori, Pacific, Asian/Other and Overall) by four points (ARCOS I 1981/1982, ARCOS II 1991/1992, ARCOS III 2002/2003, ARCOS IV 2011/2012), giving a Bonferroni-corrected p-value threshold of = 0.05/380 tests = 0.00013

**Table 2:** Crude, age-specific and age-standardised annual stroke incidence rates (first-ever strokes) per 100,000 people-years in Auckland, New Zealand across four study periods (1981–1982, 1991–1992, 2002–2003 and 2011–2012) by sex and ethnicity.

Age, sex and ethnicity group	1981–1982			1991–1992			2002–2003			2011–2012			P for
	N	n	Rate (95% CI)	N	n	Rate (95% CI)	N	n	Rate (95% CI)	N	n	Rate (95% CI)	trend
**Total**													
15–64*	518112	286	55 (46; 64)	624828	347	56 (48; 63)	788106	391	50 (45; 55)	956037	528	55 (51; 60)	
65–74	49812	260	522 (432; 612)	56388	373	661 (564; 759)	59454	336	565 (505; 626)	95190	363	381 (342; 421)	
75–84	22965	350	1524 (1298; 1750)	31701	412	1300 (1139; 1460)	37815	438	1158 (1050; 1267)	48387	442	913 (828; 999)	
85+	5691	134	2355 (1791; 2918)	8541	173	2026 (1684; 2367)	12507	258	2063 (1811; 2315)	19578	310	1583 (1407; 1760)	
Total	596580	1030	173 (158; 188)	721458	1305	181 (168; 194)	897882	1423	158 (150; 167)	1119192	1643	147 (140; 154)	<0.0001
**Standardised** ^**§**^			**156 (143; 170)**			**156 (145; 167)**			**139 (132; 147)**			**119 (114; 125)**	<0.0001
**Male**													
15–64*	256500	164	64 (50; 78)	308997	197	64 (52; 75)	380139	216	57 (49; 64)	461418	264	57 (50; 64)	
65–74	22251	158	710 (553; 867)	25452	201	790 (628; 951)	28173	198	703 (605; 801)	45678	211	462 (400; 524)	
75–84	8742	150	1716 (1328; 2104)	11946	155	1298 (1038; 1557)	15210	189	1243 (1065; 1420)	21759	223	1025 (890; 1159)	
85+	1509	38	2518 (1386; 3651)	2421	34	1404 (932; 1876)	3633	64	1762 (1330; 2193)	6807	93	1366 (1089; 1644)	
Total	289002	510	176 (155; 198)	348816	587	168 (150; 186)	427155	667	156 (144; 168)	535662	791	148 (137; 158)	0.0006
**Standardised**			**184 (163; 209)**			**167 (150; 185)**			**156 (144; 168)**			**129 (120; 138)**	**<0.0001**
**Female**													
15–64*	261612	122	47 (35; 58)	315831	150	47 (38; 57)	407967	175	43 (37; 49)	494631	264	53 (47; 60)	
65–74	27561	102	370 (269; 472)	30936	172	556 (437; 675)	31281	138	441 (368; 515)	49509	152	307 (258; 356)	
75–84	14223	200	1406 (1131; 1682)	19755	257	1301 (1096; 1505)	22605	249	1102 (965; 1238)	26634	219	822 (713; 931)	
85+	4182	96	2296 (1646; 2945)	6120	139	2271 (1833; 2709)	8874	194	2186 (1879; 2494)	12771	217	1699 (1473; 1925)	
Total	307578	520	169 (149; 190)	372642	718	193 (175; 211)	470727	756	161 (149; 172)	583545	852	146 (136; 156)	<0.0001
**Standardised** ^**§**^			**133 (118; 151)**			**143 (130; 158)**			**124 (115; 133)**			**110 (103; 119)**	**<0.0001**
**European**													
15–64*	422202	224	53 (43; 63)	459267	233	51 (42; 59)	501426	222	44 (38; 50)	450759	252	56 (49; 63)	
65–74	47481	238	501 (411; 591)	52125	341	654 (552; 756)	48633	219	450 (391; 510)	64806	239	369 (322; 416)	
75–84	22209	342	1540 (1309; 1771)	30303	387	1277 (1114; 1441)	34332	378	1101 (990; 1212)	35916	354	986 (883; 1088)	
85+	5577	130	2331 (1764; 2898)	8253	167	2024 (1675; 2372)	11790	233	1976 (1722; 2230)	16776	279	1663 (1468; 1858)	
Total	497469	934	188 (171; 205)	549948	1128	205 (189; 221)	596181	1052	176 (166; 187)	568257	1124	198 (186; 209)	0.992
**Standardised** ^**§**^			**153 (139–167)**			**150 (139; 163)**			**124 (116; 132)**			**122 (114; 130)**	**<0.0001**
**Māori**													
15–64*	52179	36	69 (37; 101)	63762	48	75 (49; 101)	77742	53	68 (50; 87)	88470	74	84 (65; 103)	
65–74	1266	6	474 (-62; 1010)	1344	3	223 (-29; 476)	2292	22	960 (559; 1361)	4452	22	494 (288; 701)	
75–84	336	4	1190 (-459; 2840)	429	8	1865 (573; 3157)	654	10	1529 (581; 2477)	1572	19	1209 (665; 1752)	
85+	51	0	0	72	2	2778 (-1072; 6628)	144	4	2778 (56; 5500)	243	2	823 (-318; 1964)	
Total	53832	46	85 (51; 120)	65607	61	93 (65; 121)	80832	89	110 (87; 133)	94737	117	123 (101; 146)	0.014
**Standardised** ^**§**^			**134 (78; 229)**			**168 (116; 241)**			**202 (157; 259)**			**156 (128; 189)**	**0.757**
**Pacific**													
15–64*	33672	20	59 (23; 96)	64506	51	79 (55; 103)	89724	66	74 (56; 91)	107688	126	117 (97; 137)	
65–74	741	10	1350 (167; 2532)	2025	21	1037 (481; 1593)	3840	47	1224 (874; 1574)	6417	43	670 (470; 870)	
75–84	213	0	0	597	12	2010 (402; 3618)	1392	24	1724 (1034; 2414)	2679	29	1082 (689; 1476)	
85+	33	0	0	108	2	1852 (-715; 4418)	246	3	1220 (-160; 2600)	582	7	1203 (312; 2094)	
Total	34659	30	87 (43; 130)	67236	86	128 (96; 160)	95202	140	147 (123; 171)	117366	205	175 (151; 199)	<0.0001
**Standardised** ^**§**^			**147 (80; 269)**			**225 (163; 310)**			**218 (183; 261)**			**197 (171; 226)**	**0.374**
**Asian & Other combined**													
15–64*	10059	6	60 (-8; 127)	37293	15	40 (13; 68)	119214	50	42 (30; 54)	309123	76	25 (19; 30)	
65–74	324	6	1852 (-244; 3947)	894	8	895 (-86; 1875)	4689	42	896 (625; 1167)	19515	58	297 (221; 374)	
75–84	207	4	1932 (-746; 4610)	372	5	1344 (166; 2522)	1437	21	1461 (836; 2086)	8220	40	487 (336; 637)	
85+	30	4	13333 (-5146; 31812)	108	2	1852 (-715; 4418)	327	7	2141 (555; 3727)	1971	22	1116 (650; 1583)	
Total	10620	20	188 (72; 305)	38667	30	78 (40; 115)	125667	120	95 (78; 113)	338829	196	58 (50; 66)	<0.0001
**Standardised** ^**§**^			**360 (185; 701)**			**158 (92; 271)**			**166 (137; 202)**			**68 (59; 79)**	**<0.0001**

N is the estimated population size and n is the number of events.

* 16–64 in 2011–2012,

^**§**^ Age-standardised to WHO world population

Between the 1981–1982 and 2011–2012 studies there were significant increases across all ethnic groups in the proportion of patients admitted to hospital within 28 days of stroke onset (28% [95% CI 26%-31%]), and in patients having neuroimaging verification of stroke pathological types (84.9% [95% CI 62.8%-100.0%]). There were also increases in patients receiving blood pressure lowering (27.0% [95% CI 24.0%-31.0%])) and antiplatelet (aspirin, clopidogrel, dipyridamole: 21.0% [95% CI 17.0%-24.0%]) medications pre-stroke (Table 1). However, there was a significant decrease in the proportion of stroke patients receiving anticoagulants pre-stroke from 2002–2003 to 2011–2012 (2.1% [95% CI 0.66%-3.5%]).

We observed that risk factors prevalence increased significantly over 30 years (Table 1), with the proportion of patients with a history of high blood pressure (≥160/95 mmHg; including people on antihypertensive medication) increasing between the first and last survey by 15.0% (95% CI 11.7%-18.4%), myocardial infarction by 12.6% (95% CI 12.8%-15.1%), and diabetes mellitus by 12.5% (95% CI 10.1%-14.9%), across all ethnic groups except Māori for high blood pressure, myocardial infarction and Type 2 diabetes mellitus, and Asian/other people for diabetes mellitus. However, there was a statistically significant decrease in the frequency of current smokers among stroke patients by 11.9% (95% CI 9.1%-14.8%), except for Pacific people in whom the smoking rate over the last decade increased by almost two times. There was also a noticeable reduction in the proportional frequency of strokes due to SAH (2.5% [95% CI 2.3%-2.6%]), and a noticeable decline in 28-day stroke case-fatality of 14.8% [95% CI 9.7%-20.0%] from 1981–1982 in 2011–2012 across all ethnic groups. Māori and Pacific people with stroke had a greater prevalence of smoking (40.4% and 24.4%) and diabetes mellitus (29.7% and 43.3%) compared with NZ Europeans (12.8% and 16.5%, respectively), but lower prevalence of MI (18.1% and 12.6% vs 28.0%) (Table 1).

Between 2002–2003 and 2011–2012, there were significant increases in the proportion of stroke patients identified as having atrial fibrillation (AF) (7.2% [95% CI 2.4%-12.0%], only significant for NZ Europeans), patients receiving lipid lowering medications (26.6% [15.0%-38.2%], patients admitted to acute stroke unit (37.3% [95% CI 18.1–56.5%]). There was a significant decrease of patients with undetermined stroke (from 11.7% to 1.9%), across all ethnic groups.

Age-standardised stroke mortality rates (Table 3) reduced by almost 3 times between 1981–1982 and 2011–2012, declining from 98/100,000 (95% CI 88/100,000–110/100,000) person-years in 1981–1982 to 37/100,000 (95% CI 34/100,000–40/100,000) person-years in 2011–2012 (test for trend p < 0.0001 [which remained significant after adjustment for multiple-testing]). Decreases in age-adjusted 1-year stroke mortality rates were seen in NZ Europeans (2% per year), Māori (1.4% per year) and Asian/other ethnic groups (7% per year) over the last 30 years. Age-adjusted stroke mortality rates (per 100,000 person-years) in Pacific people reduced from 127 (95% CI 81–198) in 1991–1992 to 124 (95% CI 96–162) in 2002–2003 and 74 (95% CI 58–95) in 2011–2012 (p for trend = 0.0002).

**Table 3:** Crude, age-specific and age-standardised annual stroke mortality rates per 100,000 people in Auckland, New Zealand across four study periods (1981–1982, 1991–1992, 2002–2003 and 2011–2012) by sex and ethnicity.

Age and ethnic group	1981–1982			1991–1992			2002–2003			2011–2012			Trend
	N	n	Rate (95% CI)	N	n	Rate (95% CI)	N	n	Rate (95% CI)	N	n	Rate (95% CI)	*P* value
**Total**													
15–64*	518112	104	20 (15; 26)	624828	122	20 (16; 23)	788106	89	11 (9; 14)	956037	84	9 (7; 11)	
65–74	49812	168	337 (265; 409)	56388	136	241 (196; 287)	59454	99	167 (134; 199)	95190	90	95 (75; 114)	
75–84	22965	266	1158 (961; 1355)	31701	226	713 (615; 811)	37815	232	614 (535; 692)	48387	184	380 (325; 435)	
85+	5691	126	2214 (1667; 2761)	8541	148	1733 (1422; 2044)	12507	227	1815 (1579; 2051)	19578	237	1211 (1056; 1365)	
Total	596580	664	111 (99; 123)	721458	632	88 (80; 95)	897882	647	72 (67; 78)	1119192	595	53 (49; 57)	<0.0001
**Standardise** ^**§**^			**98 (88; 110)**			**72 (67; 79)**			**57 (53; 62)**			**37 (34; 40)**	**<0.0001**
**Male**													
15–64*	256500	58	23 (14; 31)	308997	61	20 (15; 25)	380139	48	13 (9; 16)	461418	36	8 (5; 10)	
65–74	22251	98	440 (317; 564)	25452	83	326 (242; 410)	28173	59	209 (156; 263)	45678	55	120 (89; 152)	
75–84	8742	100	1144 (827; 1461)	11946	102	854 (670; 1038)	15210	88	579 (458; 699)	21759	96	441 (353; 529)	
85+	1509	28	1856 (884; 2828)	2421	22	909 (529; 1288)	3633	58	1596 (1186; 2007)	6807	73	1072 (826; 1318)	
Total	289002	284	98 (82; 114)	348816	268	77 (67; 87)	427155	253	59 (52; 67)	535662	260	49 (43; 54)	<0.0001
**Standardise** ^**§**^			**104 (88; 123)**			**76 (67; 87)**			**59 (52; 66)**			**39 (35; 44)**	**<0.0001**
**Female**													
15–64*	261612	46	18 (10; 25)	315831	61	19 (14; 25)	407967	41	10 (7; 13)	494631	48	10 (7; 12)	
65–74	27561	70	254 (170; 338)	30936	53	171 (125; 217)	31281	40	128 (88; 168)	49509	35	71 (47; 94)	
75–84	14223	166	1187 (916; 1418)	19755	124	628 (517; 738)	22605	144	637 (533; 741)	26634	88	330 (261; 399)	
85+	4182	98	2343 (1687; 3000)	6120	126	2059 (1651; 2466)	8874	169	1904 (1617; 2192)	12771	164	1284 (1088; 1481)	
Total	307578	380	124 (106; 141)	372642	364	98 (87; 108)	470727	394	84 (75; 92)	583545	335	57 (51; 64)	<0.0001
**Standardise** ^**§**^			**92 (79; 106)**			**67 (60; 75)**			**55 (50; 61)**			**35 (32; 40)**	**<0.0001**
**NZ European**													
15–64*	422202	76	18 (12; 24)	459267	72	16 (12; 20)	501426	44	9 (6; 11)	450759	35	8 (5; 10)	
65–74	47481	156	329 (256; 401)	52125	120	230 (183; 277)	48633	60	123 (92; 155)	64806	49	76 (54; 97)	
75–84	22209	258	1162 (961; 1362)	30303	204	673 (578; 768)	34332	193	562 (483; 641)	35916	135	376 (312; 439)	
85+	5577	122	2188 (1639; 2737)	8253	145	1757 (1437; 2076)	11790	195	1654 (1422; 1886)	16776	204	1216 (1049; 1383)	
Total	497469	612	123 (109; 137)	549948	541	98 (89; 107)	596181	492	83 (75; 90)	568257	423	74 (67; 82)	<0.0001
**Standardise** ^**§**^			**96 (86; 107)**			**67 (61; 74)**			**49 (45; 54)**			**35 (31; 39)**	**<0.0001**
**Māori**													
15–64*	52179	14	27 (7; 47)	63762	18	28 (15; 41)	77742	16	21 (10; 31)	88470	17	19 (10; 28)	
65–74	1266	6	474 (-62; 1010)	1344	3	223 (-29; 476)	2282	7	305 (79; 532)	4452	4	90 (2; 178)	
75–84	336	4	1190 (-459; 2840)	429	9	2098 (727; 3469)	654	5	765 (94; 1435)	1572	9	573 (198; 947)	
85+	51	0	0	72	2	2778 (-1072; 6628)	144	2	1389 (-536; 3314)	243	3	1235 (-162; 2632)	
Total	53832	24	45 (19; 70)	65607	32	49 (32; 66)	80832	30	37 (24; 50)	94737	33	35 (23; 47)	0.203
**Standardise** ^**§**^			**96 (47; 196)**			**133 (85; 209)**			**77 (50; 118)**			**53 (36; 77)**	**0.015**
**Pacific**													
15–64*	33672	12	36 (7; 64)	64506	27	42 (26; 58)	89724	17	19 (10; 28)	107688	22	20 (12; 29)	
65–74	741	4	540 (-208; 1288)	2025	10	494 (188; 800)	3840	21	547 (313; 781)	6417	20	312 (175; 448)	
75–84	213	0	0	597	10	1675 (135; 3215)	1392	19	1365 (751; 1979)	2679	16	597 (305; 890)	
85+	33	0	0	108	0	0	246	7	2846 (738; 4954)	582	10	1718 (653; 2783)	
Total	33	16	46 (14; 78)	67236	47	70 (48; 92)	95202	64	67 (51; 84)	117366	68	58 (44; 72)	0.949
**Standardise** ^**§**^			**69 (30; 160)**			**127 (81; 198)**			**124 (96; 162)**			**74 (58; 95)**	**0.005**
**Asian/other**													
15–64*	10059	2	20 (-19; 59)	37293	5	13 (2; 25)	119214	11	9 (4; 15)	309123	10	3 (1; 5)	
65–74	324	2	617 (-593; 1827)	894	3	336 (-44; 715)	4689	8	171 (52; 289)	19515	16	82 (42; 122)	
75–84	207	4	1932 (-746; 4610)	372	3	806 (-106; 1719)	1437	10	696 (265; 1127)	8220	24	292 (175; 409)	
85+	30	4	13333 (-5146; 31812)	108	1	926 (-889; 2741)	327	8	2446 (751; 4142)	1971	20	1015 (570; 1459)	
Total	10620	12	113 (23; 203)	38667	12	31 (13; 49)	125667	37	29 (20; 39)	338829	70	21 (16; 25)	<0.0001
**Standardise** ^**§**^			**238 (102; 557)**			**70 (37; 132)**			**64 (45; 91)**			**27 (21; 34)**	**<0.0001**

* 16–64 in 2011–2012,

^**§**^ Age-standardised to WHO world population

**Fig 1:**
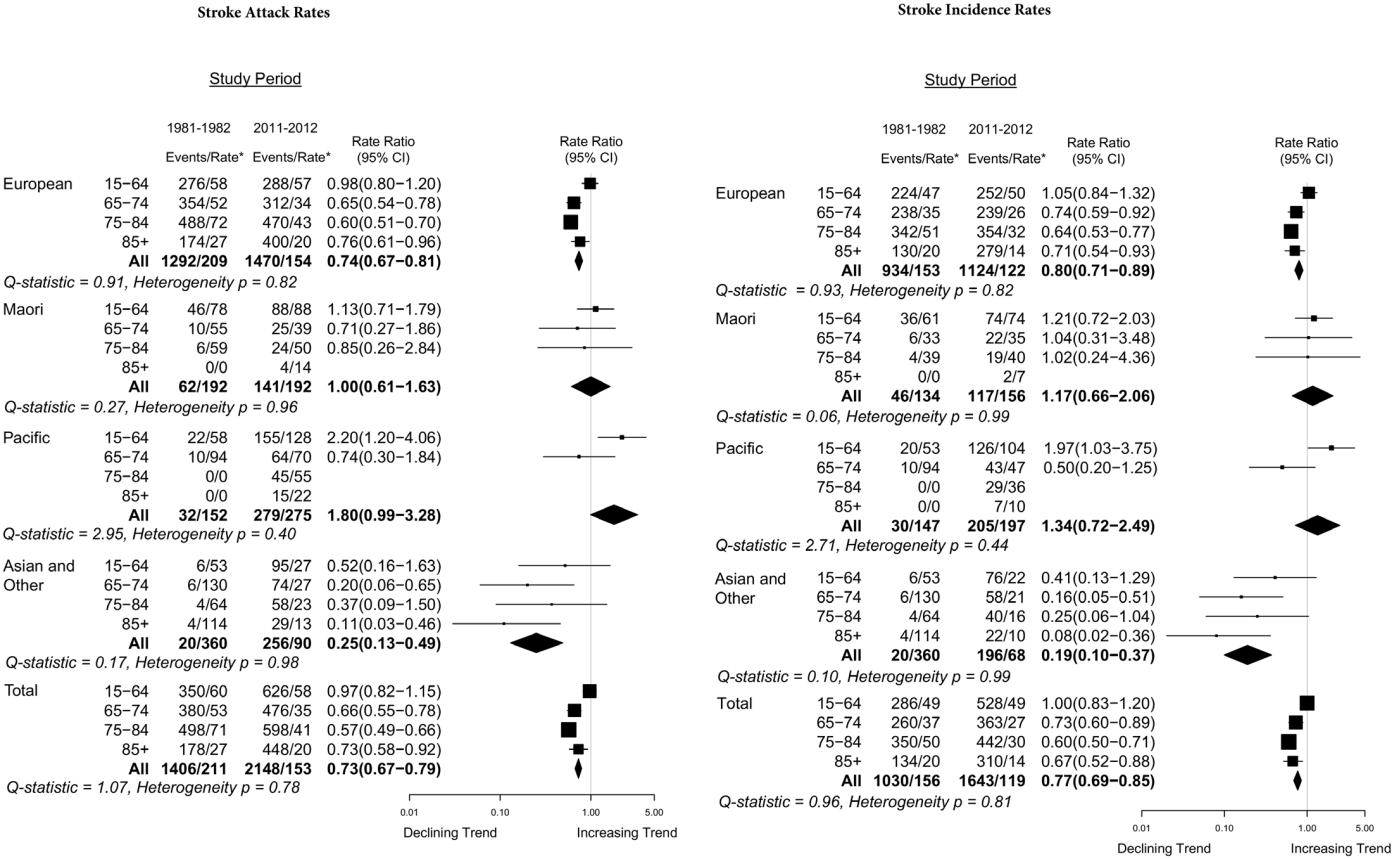
Forest plot of stroke incidence and attack rate ratios (2011–2012 compared with 1981–1982), with rates age-adjusted to the WHO world population.

**Fig 2:**
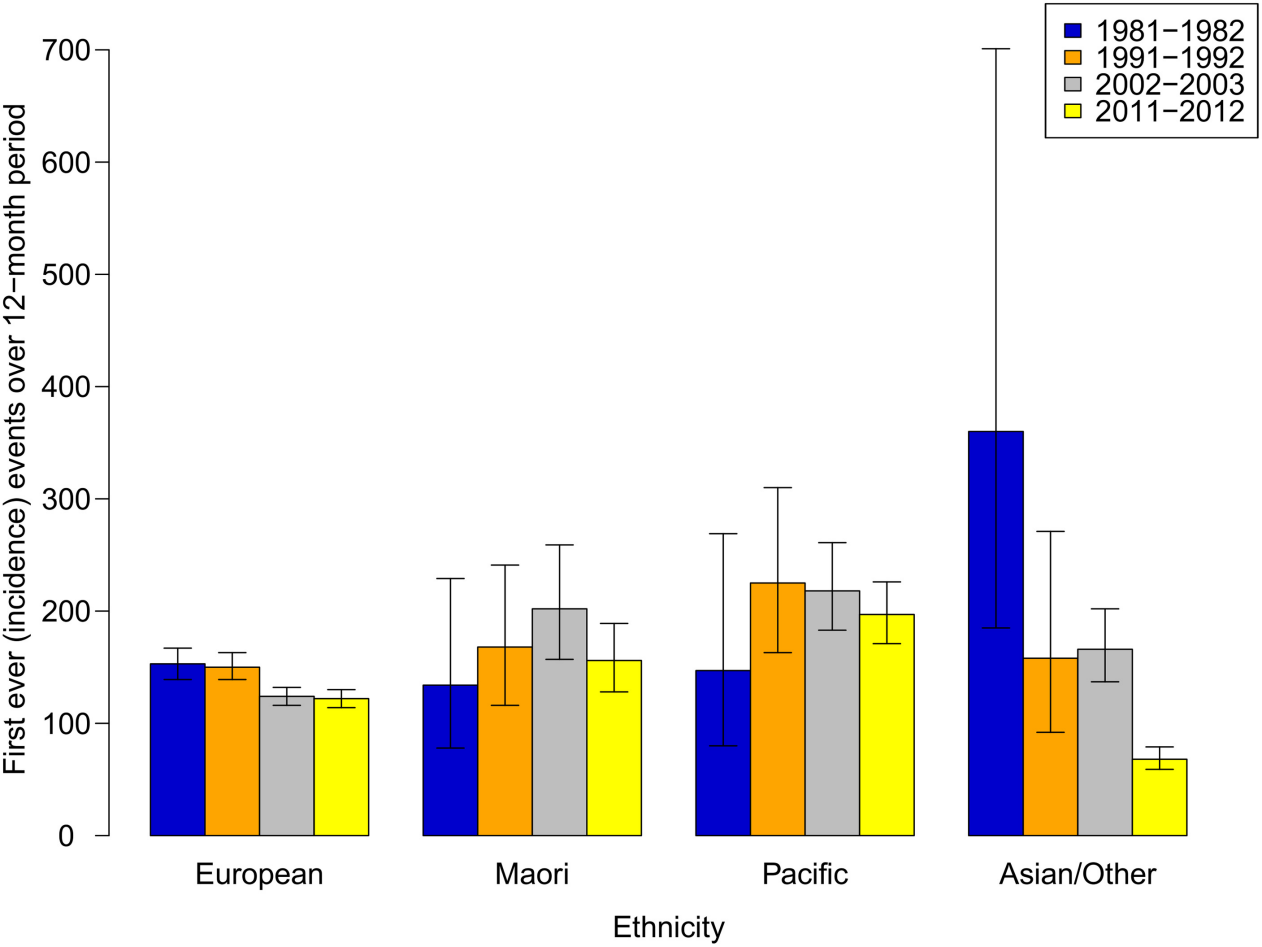
Stroke incidence rates over 3 decades by ethnicity.

**Fig 3:**
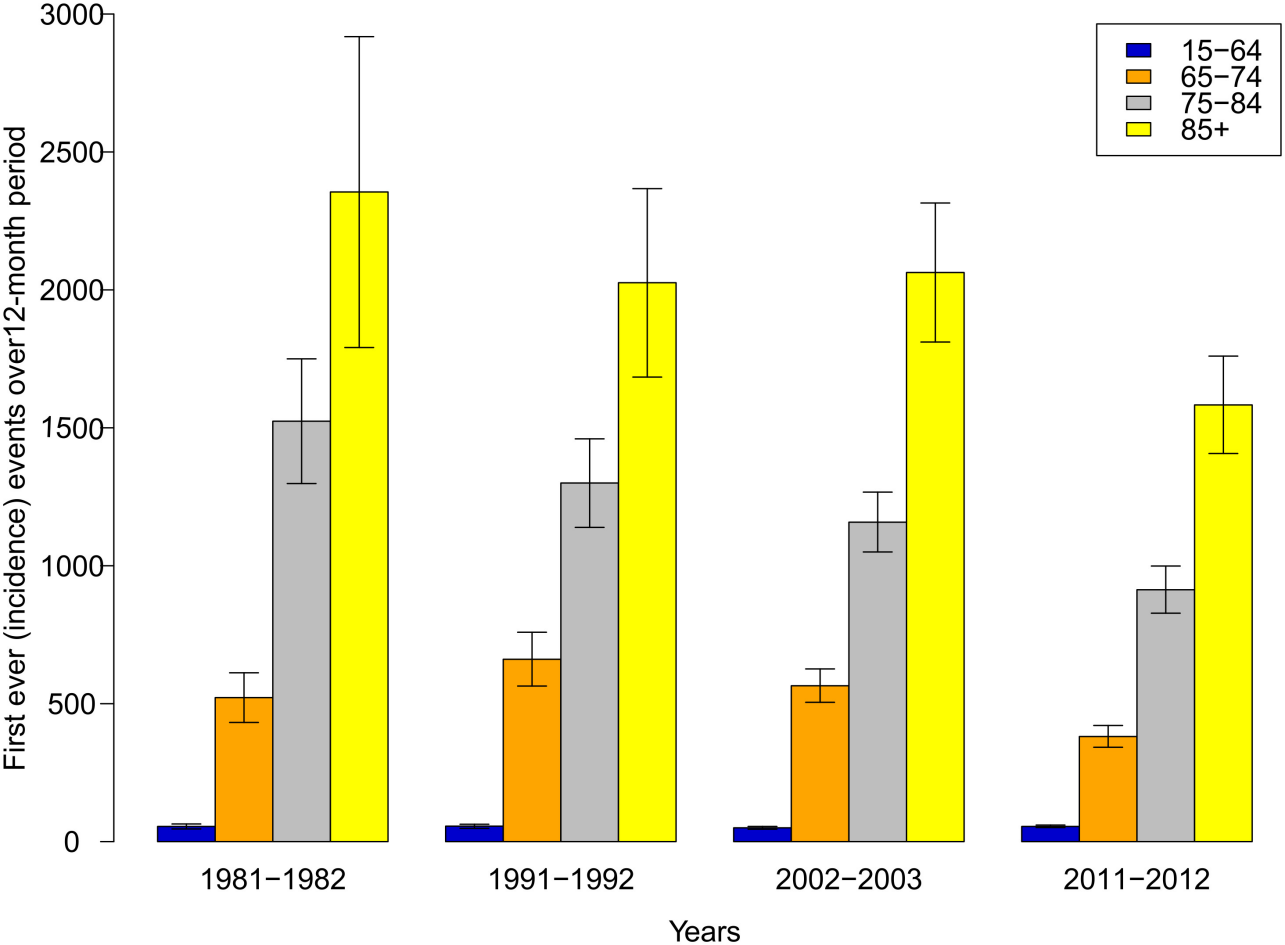
Stroke incidence rates over 3 decades by 4 age groups.

Over the 30-year study period, there was a 23% (95% CI 15%-31%) reduction in stroke incidence (Fig 1) and 27% (95% CI 21%-33%) reduction in stroke attack rate overall (Table 4 and Fig 1). These reductions were largely due to decreases in NZ Europeans (decrease 20% in incidence, 26% attack rates) and Asian/others (decrease 81% [95% CI 63%-90%] in incidence, 75% [95% CI 51%-87%] in attack rates) (Figs 2 and 3). However, in Māori and Pacific people, there were non-significant increases in stroke incidence (first-ever strokes) and attack rates (incident and recurrent strokes combined) between 1981–1982 and 2011–2012 study periods. Overall, decreases in stroke incidence and attack rates were limited to people 65 years or older (Fig 3). In people aged 15–64 years, there were no significant changes in stroke incidence or attack rates, except for Pacific people where stroke incidence and attack rates doubled (Fig 1).

**Table 4:** Crude, age-specific and age-standardised annual attack (first-ever and recurrent strokes combined) rates per 100,000 people-years in Auckland, New Zealand across four study periods (1981–1982, 1991–1992, 2002–2003 and 2011–2012) by sex and ethnicity.

Age, sex and ethnicity group	1981–1982			1991–1992			2002–2003			2011–2012			*P* value for trend
	N	n	Rate (95% CI)	N	n	Rate (95% CI)	N	n	Rate (95% CI)	N	n	Rate (95% CI)	
**Total**													
15–64*	518112	350	68 (58;78)	624828	433	69 (61; 77)	788106	484	61 (56; 67)	956037	626	65 (60; 71)	
65–74	49812	380	763 (654; 871)	56388	512	908 (795; 1021)	59454	460	774 (703; 844)	95190	476	500 (455; 545)	
75–84	22965	498	2169 (1899; 2438)	31701	611	1927 (1730; 2125)	37815	667	1764 (1630; 1898)	48387	598	1236 (1137; 1335)	
85+	5691	178	3128 (2478; 3778)	8541	247	2892 (2460; 3324)	12507	390	3118 (2809; 3428)	19578	448	2288 (2076; 2500)	
Total	596580	1406	236 (218; 253)	721458	1803	250 (235; 265)	897882	2001	223 (213; 233)	1119192	2148	192 (184; 200)	<0.0001
**Standardised** ^**§**^			**211 (196; 228)**			**213 (201; 227)**			**193 (185; 202)**			**153 (147; 160)**	**<0.0001**
**Male**													
15–64*	256500	204	80 (64; 95)	308997	252	82 (69; 94)	380139	273	72 (63; 80)	461418	323	70 (62; 78)	
65–74	22251	224	1007 (820; 1193)	25452	290	1139 (947; 1332)	28173	273	969 (854; 1084)	45678	272	595 (525; 666)	
75–84	8742	216	2471 (2005; 2937)	11946	252	2109 (1768; 2451)	15210	277	1821 (1607; 2036)	21759	305	1402 (1244; 1559)	
85+	1509	46	3048 (1803; 4294)	2421	41	1694 (1175; 2212)	3633	95	2615 (2089; 3141)	6807	138	2027 (1689; 2366)	
Total	289002	690	239 (214; 264)	348816	835	239 (218; 261)	427155	918	215 (201; 229)	535662	1038	194 (182; 206)	<0.0001
**Standardised** ^**§**^			**248 (223; 276)**			**236 (216; 258)**			**214 (200; 228)**			**167 (157; 178)**	**<0.0001**
**Female**													
15–64*	261612	146	56 (43; 69)	315831	181	57 (47; 67)	407967	211	52 (45; 59)	494631	303	61 (54; 68)	
65–74	27561	156	566 (440; 692)	30936	222	718 (587; 848)	31281	187	598 (512; 683)	49509	204	412 (356; 469)	
75–84	14223	282	1983 (1655; 2310)	19755	359	1817 (1577; 2058)	22605	390	1725 (1554; 1897)	26634	293	1100 (974; 1226)	
85+	4182	132	3156 (2395; 3918)	6120	206	3366 (2799; 3934)	8874	295	3324 (2945; 3704)	12771	310	2427 (2157; 2698)	
Total	307578	716	233 (209; 257)	372642	968	260 (239; 281)	470727	1083	230 (216; 244)	583545	1110	190 (179; 201)	<0.0001
**Standardised** ^**§**^			**181 (163; 201)**			**190 (175; 206)**			**173 (162; 184)**			**140 (132; 149)**	**<0.0001**
**NZ European**													
15–64*	422202	276	65 (54; 76)	459267	293	64 (55; 73)	501426	269	54 (47; 60)	450759	288	64 (57; 71)	
65–74	47481	354	746 (636; 855)	52125	459	881 (764; 997)	48633	303	623 (553; 693)	64806	312	481 (428; 535)	
75–84	22209	488	2197 (1922; 2473)	30303	579	1911 (1709; 2113)	34332	568	1654 (1518; 1790)	35916	470	1309 (1190; 1427)	
85+	5577	174	3120 (2464; 3776)	8253	241	2920 (2476; 3364)	11790	345	2926 (2617; 3235)	16776	400	2384 (2151; 2618)	
Total	497469	1292	260 (240; 280)	549948	1572	286 (267; 305)	596181	1485	249 (236; 262)	568257	1470	259 (245; 272)	0.165
**Standardised** ^**§**^			**209 (193; 226)**			**206 (193; 220)**			**171 (162; 180)**			**154 (145; 163)**	**<0.0001**
**Māori**													
15–64*	52179	46	88 (52; 124)	63762	58	91 (63; 119)	77742	60	77 (58; 97)	88470	88	99 (79; 120)	
65–74	1266	10	790 (98; 1482)	1344	8	595 (183; 1008)	2282	24	1047 (628; 1466)	4452	25	562 (341; 782)	
75–84	336	6	1786 (-235; 3806)	429	14	3263 (1554; 4973)	654	15	2294 (1133; 3454)	1572	24	1527 (916; 2138)	
85+	51	0	0	72	2	2778 (-1072; 6628)	144	5	3472 (429; 6516)	243	4	1646 (33; 3259)	
Total	53832	62	115 (75; 156)	65607	104	129 (104; 153)	80832	104	129 (104; 153)	94737	141	149 (124; 173)	0.073
**Standardised** ^**§**^			**192 (122; 304)**			**254 (188; 341)**			**247 (196; 311)**			**192 (161; 229)**	**0.867**
**Pacific**													
15–64*	33672	22	65 (27; 104)	64506	65	101 (74; 127)	89724	88	98 (78; 119)	107688	155	144 (121; 167)	
65–74	741	10	1350 (167; 2532)	2025	33	1630 (899; 2360)	3840	67	1745 (1327; 2163)	6417	64	997 (753; 1242)	
75–84	213	0	0	597	13	2178 (536; 3819)	1392	39	2802 (1922; 3661)	2679	45	1680 (1189; 2171)	
85+	33	0	0	108	2	1852 (-715; 4418)	246	8	3252 (998; 5506)	582	15	2577 (1273; 3882)	
Total	34659	32	92 (47; 138)	67236	113	168 (131; 205)	95202	202	212 (183; 241)	117366	279	238 (210; 266)	<0.0001
**Standardised** ^**§**^			**152 (85; 274)**			**291 (220; 384)**			**329 (284; 382)**			**275 (244; 310)**	**0.012**
**Asian/other**													
15–64*	10059	6	60 (-8; 127)	37293	17	46 (17; 74)	119214	64	54 (41; 67)	309123	95	31 (25; 37)	
65–74	324	6	1852 (-244; 3947)	894	12	1342 (268; 2416)	4689	55	1173 (863; 1483)	19515	74	379 (293; 466)	
75–84	207	4	1932 (-746; 4610)	372	5	1344 (166; 2522)	1437	34	2366 (406; 3161)	8220	58	706 (524; 887)	
85+	30	4	13333 (-5146; 31812)	108	2	1852 (-715; 4418)	327	10	3058 (1163; 4954)	1971	29	1471 (936; 2007)	
Total	10620	20	188 (72; 305)	38667	36	93 (54; 132)	125667	163	130 (110; 150)	338829	256	76 (66; 85)	<0.0001
**Standardised** ^**§**^			**360 (185; 701)**			**194 (122; 310)**			**234 (197; 277)**			**90 (79; 101)**	**<0.0001**

* 16–64 in 2011–2012,

^§^ Age-standardised to WHO world population

In 2011–2012, 54% (95% CI 48%-61%) of first ever incident and 51% (95% CI 46%-57%) of recurrent strokes occurred in people younger than 75 years (Table 2). The proportion of incident and recurrent strokes in Māori and Pacific people younger than 75 years was twice that of NZ Europeans (44% [95% CI 40%-48%] and 41% [95% CI 35%-47%] incident and recurrent stroke in NZ Europeans aged 15–74 years compared to 82% [95% CI 74%-90%] and 80% [95% CI 72%-89%] in Māori and 82% [95% CI 73%-93%] and 78% [95% CI 69%-89%] in Pacific people aged 15–74 years, respectively). The temporal trends in declining stroke incidence and attack rates were consistent in both males and females aged 65 years and older (Tables 2 and 4), with the most noticeable decline observed in males (Tables 2 and 4) and NZ Europeans (Tables 2 and 4, Fig 1).

**Fig 4:**
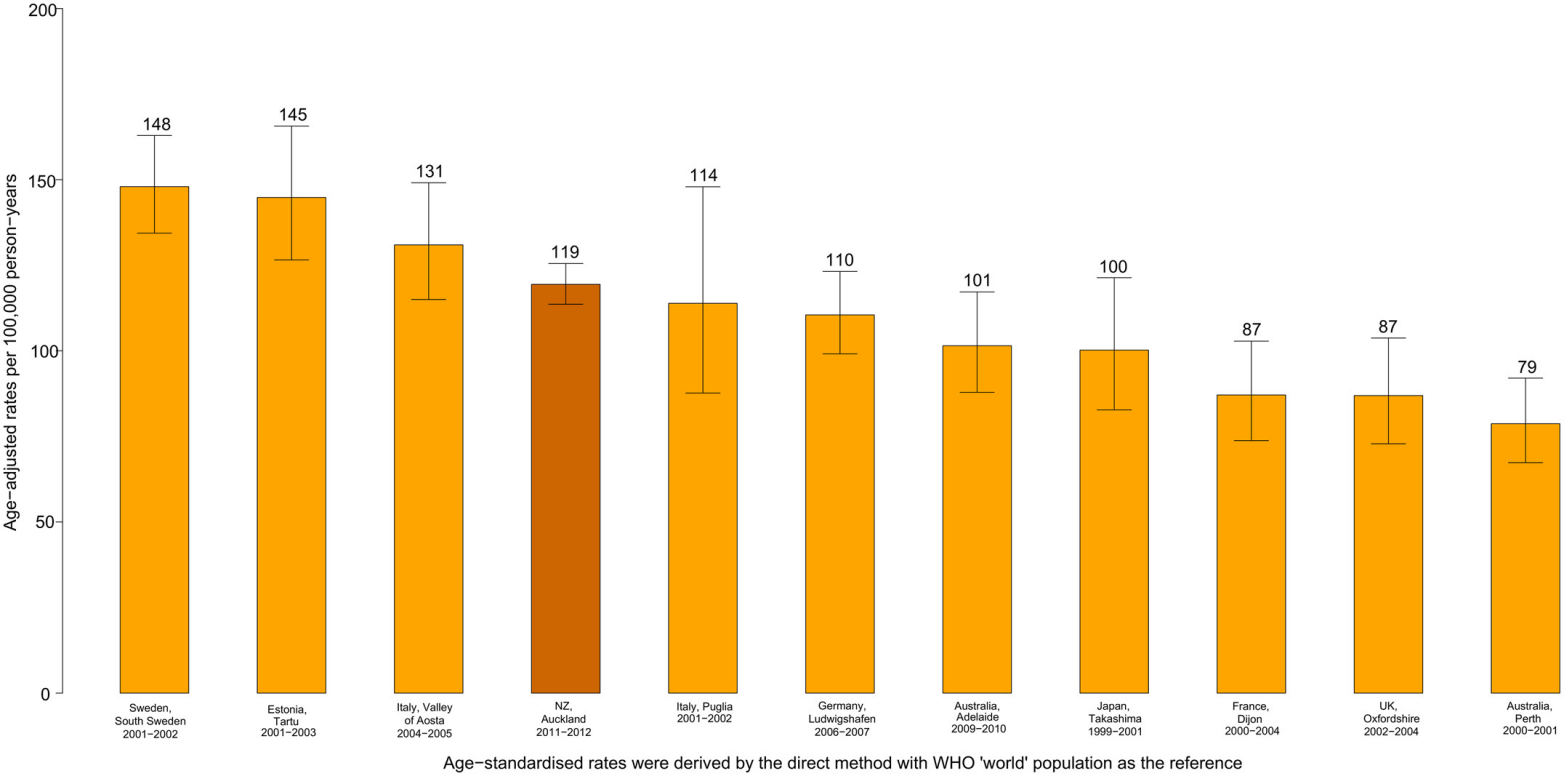
Annual age-adjusted stroke incidence rates in population-based studies[14,26–32,48–51] carried out in high-income countries in 2000–2014.

Stroke mortality rates increased with age across all study periods. In 2011–2012, there were no significant differences in age-adjusted stroke mortality rates (per 100,000 person-years) between males and females (39 [95% CI 35–44] and 35 [95% CI 32–40], respectively). In 2011–2012, age-adjusted stroke mortality rates were highest in Pacific and Māori people (74 [95% CI 58–95] and 53 [95% CI 36–77], respectively), and lowest in Asian/other and NZ/European people (27 [95% CI 21–34] and 35 [95% CI 31–39] respectively). Māori and Pacific people had a test for trend of p<0.0001, which reflected both an increase in stroke incidence and mortality rates from 1981–1992 and a decline in stroke incidence and mortality rates from 2002–2012. All other ethnicities (NZ/Europeans, Asian/other), total, males, females all had test for trend p<0.0001 which reflected the continuous decline in stroke incidence and mortality rates across the study period. The overall current level of stroke incidence rates in NZ remains high compared to other developed countries where comparable population-based stroke incidence studies were carried out in 2000–2014 period[26–32] (Fig 4).

## Discussion

The main findings of this study include: (a) a clear trend towards reducing stroke incidence, 28-day case-fatality and 1-year mortality rates; (b) an increase in the frequency of elevated blood pressure, myocardial infarction, Type 2 diabetes mellitus and AF among stroke patients, but decrease in the frequency of current smoking; (c) better survival of acute stroke patients across all ethnic groups; and (d) persistent differences in stroke incidence, mortality rates and risk factor prevalence across the four major ethnic groups in NZ. Overall, the ethnic disparities have lessened overtime, especially over the last study decade. Our findings of decrease in stroke incidence and mortality rates overall in the study population but an increase, although not significant, in stroke rates in people younger than 64 years old (particularly females, Māori and Pacific Island people) are concordant with those of the GBD 2010 Study which identified a continuous decline in stroke incidence and mortality rates in high-income countries,[1] and a trend towards a stable or increasing stroke burden in people younger than 65 years.[1] The latter is of particular concern as our data showed an increased frequency of several major vascular risk factors in people with stroke that was also observed in the general NZ population and other countries.[14,33–35] This includes an epidemic of obesity and Type 2 diabetes mellitus in children and young adults,[36,37] as well as behavioural and dietary risk factors.[38–40] An increase (or no reduction) in the incidence of stroke in middle-age populations has been observed in other studies,[41,42] thus re-emphasising the need for intensification of appropriate primary stroke prevention strategies, especially on at a population level.

Although the overall decline in stroke incidence rates is in line with some population-based studies,[43–47] the decline in stroke incidence in NZ over the last 30 years is almost 20% less than that of comparable studies for a similar study period (Fig 4).[13,14,26–32,48–51] Further, this study has highlighted that the relatively high level of stroke incidence in NZ is largely attributed to high rates in Māori and Pacific people. Moreover, unlike similar studies,[14,30,44] we found a significant trend towards increase in the prevalence of elevated blood pressure, history of myocardial infarction and diabetes mellitus among stroke patients and this may be a contributing factor to the observed differences in NZ compared to other high-income countries. Indeed, the 2008/2009 NZ Adult Nutrition Survey showed that there was an increase in mean systolic blood pressure in the overall population since 2002, but in particular in Māori men aged 35–74. The prevalence of hypertension in the NZ population in 2008/2009 was estimated at 31%, with only 15% on anti-hypertensive medication.[39] However, the increasing prevalence of elevated blood pressure, MI and Type 2 diabetes in stroke patients may also be related to increased detection of undiagnosed hypertension, diabetes and other health conditions over the study period, therefore these data should be interpreted with caution. The proportion of recurrent strokes has shown a statistically significant decrease over the study period overall and NZ European and Maori people in particularly, although the level of recurrent strokes (21.4%) in NZ remains relatively high compared to some other developed countries.[52] There was no statistically significantly change in the proportion of recurrent strokes in Pacific, Asian and other ethnic groups in NZ from 1981 to 2012 and but showed a trend towards increasing. This and the overall high rate of recurrent strokes in NZ (especially in Maori and Pacific people younger than 75 years) signify the need for better, culturally appropriate secondary stroke prevention strategies.

The younger age of stroke onset in Māori is also reflected in the lower life expectancy at birth of Māori, which in 2006 was at least eight years less than that for non-Māori for both genders.[53] Of note is the increase in the prevalence of atrial fibrillation and low levels of pre-morbid anticoagulation of patients with AF over the last decade. Despite 36.1% of ischaemic stroke patients having AF in the 2011–2012 study, only 26.5% of these patients were being treated with anticoagulation therapy prior to stroke. A similar increase in the prevalence of AF in stroke patients was recently observed in another population-based study.[54] This, together with the global epidemic of AF,[55] suggests that it is now becoming the leading risk factor for stroke.

Similar diverging trends and ethnic disparities in stroke incidence have been shown in other comparable population-based studies in the US[43] and UK,[44] suggesting unfavorable trends in the prevalence of risk factors.[56] In the Atherosclerosis Risk in Communities cohort there was a 40% increase in the proportion of hypertension and diabetes from 1987 to 2011.[46] Ethnic differences in stroke risk have been attributed to differences in socioeconomic status (SES) and exposure to risk factors.[57] Lower SES groups have greater exposure to cardiovascular risk factors, including hypertension, smoking, poor diet, physical inactivity, diabetes and excess alcohol use.[58] Also, lower SES groups may have limited access to, or make less effective use of, services to manage these risk factors (e.g., early hypertension detection and control).[59] However, there is evidence that not all differences among ethnic groups are explained by differences in cardiovascular risk factors, suggesting genetic[60] and other factors such as socioeconomic disparities and the experience of discrimination[61] may be important. There remains uncertainty about the relative importance of stroke risk factor management and other factors in causing these inequalities. The impact of SES, changing family dynamics and cultural values on stroke risk and recovery in Māori and Pacific Island people is uncertain.[62] Ethnic-specific differences in stroke risk factors have also been shown in some other multi-ethnic populations[63–70] suggesting the need for culturally tailored primary and secondary preventative strategies.

If the current temporal trend in the distribution of stroke risk factors in the population continues, we are likely to observe an increase in the incidence of stroke in the near future. Primary stroke prevention is a mainstream strategy to reduce stroke burden in a population.[71–73] However, the relative significance of various determinants of stroke occurrence as well as cultural appropriateness of stroke prevention strategies, especially among Māori and Pacific people,[3] are not known, hindering the development of effective, evidence-based primary stroke prevention strategies on both individual and population levels.[4,74–79] Furthermore, a detailed analysis of recent changes in stroke risk factors is needed to explain the diverging trends in stroke incidence and mortality rates in the different ethnic groups in NZ and to further inform culturally appropriate primary stroke prevention strategies.[80]

Better management (including enhanced admission rates to acute stroke units) and resulting improved 28-day and 1-year survival of acute stroke patients have been observed in other studies.[13,32,81,82] Although hospital admission has improved in this study over the 30 year period, there is still an underutilisation of acute stroke units (only 51% of stroke patients were treated in acute stroke units) and thrombolytic therapy for ischaemic stroke patients (only 5% of ischaemic stroke patients received alteplase [data not shown]) against the recommended corresponding figures in the NZ guidelines.[83] A recent individual-participant data meta-analysis of alteplase[84] demonstrated that irrespective of age or stroke severity, and despite an increased risk of fatal intracranial haemorrhage during the first few days after treatment, alteplase significantly improves the overall odds of a good stroke outcome when delivered within 4·5 h of stroke onset, with earlier treatment associated with bigger proportional benefits. Similar to some previous observations[85,86] but contrary to others[87–89] we found a moderate, though non-statistically significant, decrease in the incidence of subarachnoid haemorrhage that may be related to the reduced smoking rates[90] in NZ.[91]

The observed level and magnitude in stroke mortality decline in Auckland, NZ (3-fold decline [from 98/100,000 person-years in 1981–1982 to 37/100,000 person-years in 2011–2012]) is similar to official NZ statistics on stroke mortality rates,[92] supporting the validity of our data. However, our findings of faster declines in stroke mortality rates than stroke incidence rates, in line with our previous estimates,[93] suggest a further rise of stroke related disability over the next decade due to population growth and aging in NZ. This is of particular importance for Māori and Pacific groups where there has been a substantial increase in stroke in younger age groups, thus emphasizing the importance and priority of primary stroke prevention. A mismatch in temporal trends between stroke incidence and mortality rates has been shown in some other populations.[94]

### Novelty and Translation

To the best of our knowledge, this is the first, longest and largest population-based survey to compare long-term trends and 1-year outcomes of stroke. There are several important new findings reported in this paper. The first is the diverging and changing trends in stroke incidence and stroke risk factors in different ethnic groups of the population. The trends in stroke incidence for Māori and Pacific Island people behave are similar to those observed in low to middle-income countries, while in NZ/ Europeans, trends in stroke incidence are like those in high-income countries with ethnically more homogeneous populations. Ethnicity/race is a proxy for disparity[95] and this is relevant internationally because other ethnically diverse societies will need to make provisions for population sub-groups prone to disparity to ensure that they have access to appropriate health care. Second, our findings were obtained before and during contemporary changes in prevention (primary and secondary) and service delivery (acute stroke unit care and thrombolysis) in a whole population context. This provides, for the first time, direct feedback to health-policy decision-makers on the relative success of these changes in medical practice. Specifically, the study demonstrated that current stroke preventative and management strategies are far more effective in reducing stroke risk and mortality on a population level than those used before mid-1990s. This should encourage health systems worldwide to seriously consider implementing modern stroke preventive and treatment strategies if they have not already done so. Third, this is the first documentation, to the best of our knowledge, of stroke mortality rates falling faster than stroke incidence rates. If this trend is also borne out in other populations then health systems will need to adapt in order to accommodate the likely increase in rehabilitation services needs in order to cater for this increase in prevalence and, therefore, make plans to implement more effective and culturally appropriate primary stroke prevention strategies at a population level. The differences in the stroke incidence trends by ethnicity are also important to take into account when projecting stroke burden in multi-ethnic communities on a national and global scale (including Global Burden of Disease estimates) and further reinforce the need for culturally appropriate prevention and health care strategies in multi-ethnic communities. The diverging trends in stroke incidence in the different ethnic groups and different rates of reduction in stroke mortality and incidence rates should be generalizable to other ethnically diverse health-care systems that have achieved similar risk factor modification.

### Strength and limitations

The strength of our study is that it includes four large prospective population-based stroke registers (5400 stroke patients; 3,335,112 person-years of observation) across a long timespan (over 30 years: 1981–2012). The major limitation of the study was a relatively low number of strokes in some ethnic groups, especially in the Asian/other ethnic group making it difficult to determine whether trends in incidence, attack and mortality rates were statistically significant. Although the numbers were small, one possible explanation for the apparent large reduction in stroke incidence and mortality rates in Asian/other ethnic group may be related to the large influx of younger, presumably generally healthy, Asian immigrants in NZ that has been particularly marked over the last two decades.[21] The NZ Health and Disability sector uses the NZ Health Statistics definition for ethnicity ‘as a social construct of group affiliation and identity’ where ‘‘ethnicity is the ethnic group or groups that people identify with or feel they belong to’.[96] In the 1981–1982 and 2002–2003 studies, only one ethnicity was collected for participants in each study, preventing us from applying prioritised ethnicity classification in these studies. Although this may have introduced a classification bias by ethnicity, we believe the effect of the bias was not large and was likely to be non-differential as most of the ethnic priority classifications in the 1991–1992 and 2011–2012 studies were also based on the first ethnicity self-identified by the study participants. In addition, less than 10% of the last surveys study participants indicated more than one ethnicity. We lacked statistical power to stratify our analyses by ethnicity to generate specific ethnicity-covariate estimates for risk of stroke onset due to having small numbers of stroke patients per ethnic minority, particularly the Asian subgroup. Given the continuous population increase in Auckland and in New Zealand in general, it is likely that we will have improved statistical power to investigate ethnicity-covariate interactions in a future ARCOS study. Another limitation of our series of studies is that the first two ARCOS studies (1981–1982 and 1991–1992) used a cluster sampling scheme approach for case-ascertainment[97] and, therefore, may have underestimated the number of stroke cases in these study periods. Although we applied the same criteria for cardiovascular risk factors recorded across all studies, we acknowledge that the accuracy of information about these risk factors was prone to some degree of variation and, therefore, should be interpreted with caution. However, the consistency of the assessment procedures, diagnostic criteria, the implementation of quality control procedures during the four studies, together with the decreased estimated number of missing stroke cases (as determined by capture-recapture analyses), reassure us that case-ascertainment was high across all these studies. Finally, due to the lack of reliable verification of pathological types of stroke in the 1981–1982 and 1991–1992 studies, analysis of changes in pathological types of stroke was limited to the 2002–2003 and 2011–2012 ARCOS studies. The larger proportion of ischaemic strokes in 2011–2012 compared to 2002–2003 may be related to a greater use of brain scanning (97% in 2011–2012; 88% in 2002–2003) which shifted classification of some strokes as ‘undetermined’ to the ischaemic group.

## Conclusions

A continuous and recently accelerated reduction in total stroke incidence and mortality has been found over the last three decades in the Auckland region of NZ. Despite a trend towards closing the gap in reducing the difference in stroke burden between NZ European and non-European ethnic groups in NZ, the 15-year differences in the mean age of stroke onset in Māori and Pacific compared to NZ/ Europeans has persisted. Some positive changes in the profile of risk factors in people with stroke, such as overall decline in the proportion of current smokers and recurrent strokes, and increased use of antiplatelet, blood pressure and lipid lowering medications, were counterbalanced by increasing prevalence of high blood pressure, myocardial infarction, diabetes mellitus, AF, and low use of anticoagulants in people with AF. This, together with the ongoing discordance between trends in stroke incidence and mortality rates and persistent ethnic disparities in stroke burden, emphasises the need for better understanding of causes of ethnic disparities in stroke burden. Culturally appropriate primary stroke prevention strategies need to be prioritised at a population level.[98] Some of these strategies need government intervention in terms of increases in taxes on cigarettes and alcohol for example, reduction in the levels of salt and sugar in the population,[99,100] the use of advertising campaigns informed by behavioural change methods to reduce smoking, alcohol, increase physical activity, or evidence-based interventions. Government led health interventions should include stroke programmes ideally already at the school age in the regular teaching plan among other health education programs. To inform future stroke prevention strategies and reduce stroke burden, a better understanding (including modelling)[101] of the interplay between trends in stroke (and stroke sub-types) risk factors, demographic (e.g., aging of the population, ethnic/racial disparities) and health care (e.g., introduction of acute stroke units, thrombolysis for ischaemic stroke), morbidity (e.g. incidence and disability) and mortality rate changes in different populations is required.
